# Molecular Dynamics Calculation on the Adhesive Interaction Between the Polytetrafluoroethylene Transfer Film and Iron Surface

**DOI:** 10.3389/fchem.2021.740447

**Published:** 2021-09-23

**Authors:** Zhen Zuo, Lifen Liang, Qianqian Bao, Pengtao Yan, Xin Jin, Yulin Yang

**Affiliations:** ^1^ School of Mechanical Engineering, Beijing Institute of Technology, Beijing, China; ^2^ Aviation Key Laboratory of Science and Technology on Generic Technology of Self-Lubricating Spherical Plain Bearing, Yanshan University, Qinhuangdao, China; ^3^ College of Mechanical Engineering, Yanshan University, Qinhuangdao, China; ^4^ School of Physics and Electronic Engineering, Xingtai University, Xingtai, China

**Keywords:** molecular dynamics, adsorption, polytetrafluoroethylene, transfer film, interfacial interaction

## Abstract

During the friction process, the polytetrafluoroethylene (PTFE) adhered on the counterpart surface was known as the PTFE transfer film, which was fundamental to the lubricating performance of the PTFE. However, the adhesive interaction between the iron surface and the adhered PTFE transfer film is still unclear. In present study, molecular dynamics simulations were used to reveal the adhesive interaction between the iron surface and PTFE transfer film. Based on the atomic trajectories obtained through the molecular dynamics, the interaction energy, concentration profile, radial distribution function, and mean square displacement were calculated to analyze the structure of the interface. The negative values of the interaction energy demonstrated the adhesive interaction between the PTFE transfer film and Fe surfaces, resulting in the accumulation of the PTFE transfer film on the Fe surface. Among the (100) (110), and (111) surfaces of α-Fe (110) surface owns the strongest adhesive interaction with the PTFE transfer film. Compared with the original PTFE molecule, the chain broken PTFE, hydroxyl substituted PTFE, and carbonyl substituted PTFE exhibited stronger adhesive interaction with Fe surface. The adhesive interaction between the PTFE transfer film and Fe surfaces was mainly originated from the Fe atoms and the F atoms of the adsorbate PTFE transfer film, which was governed by the van der Waals force. The bonding distance between the Fe atom and the F atom of the adsorbate PTFE transfer film is around 2.8 Å. Moreover, the chain broken of PTFE molecule and the rise of temperature can remarkably increase the mobility of polymer chains in the interface system.

## Introduction

Multifarious polymers are widely used in the field of self-lubricating. As one of the famous self-lubricating polymers, polytetrafluoroethylene (PTFE) owns the low coefficient of friction originated from the PTFE transfer film (Yeo and Polycarpou, 2014; Zhang et al., 2009; Zuo et al., 2015a), which is defined as the polymer molecules transferred onto the metal counterpart surface during the friction process. Therefore, the investigation on the formation of PTFE transfer film can be helpful to reveal the self-lubricating mechanism of PTFE. The component of composites and the morphology of PTFE transfer film has direct impacts on the tribological performances of PTFE composites. Researchers found that certain fillers can not only enhance the antiwear property of PTFE, but also be more conducive to the formation of PTFE transfer film (Wang and Yan, 2006; Bahadur, 2000; Friedrich et al., 2005; Unal et al., 2004). The PTFE transfer film with the thin and uniform morphology exhibited better antifriction property than that of the thick and uneven morphology (Xie et al., 2010; Ye et al., 2013).

With the rapid development of computational techniques, molecular dynamics simulation has now developed as an effective tool to be utilized in the tribological field, which can provide more information at the atomic level (Ewen et al., 2018). Molecular dynamics simulations were used to study the relationship between the molecular structure and the tribological property of PTFE (Jang et al., 2007; Chiu et al., 2011). Pan et al. (2019) analyzed the influence of the normal pressure on the friction performance of PTFE. Also, the formation of the PTFE transfer film on the Al2O3 surface was investigated by the molecular dynamics simulations (Onodera et al., 2013; Onodera et al., 2014; Onodera et al., 2017). However, to the best of our knowledge, the adhesion mechanism of PTFE transfer film on the Fe surface has not been reported.

To date, a large number of molecular dynamics calculations were applied to evaluate the interfacial interaction of polymers. For instance, the interfacial adhesive interaction between the graphene oxide and calcium silicate hydrate was calculated by the molecular dynamics (Wang et al., 2020). Liu et al. (2015) investigated the interfacial interaction between the graphene and two type of polymers (polyethylene, polymethyl methacrylate). Molecular dynamics calculations were utilized to evaluate the interfacial properties of the epoxy composites containing the oxygen-functionalized graphene (Yang et al., 2019). Johnston et al. performed molecular dynamics simulations to study the interaction between the carbon fiber and DGEBF epoxy resin substrate (Johnston et al., 2017). Using the molecular dynamics simulations, Moon et al. (2017) analyzed the interfacial strengthening mechanism between the graphene and polypropylene.

This study aims to illustrate the adhesive interaction between the iron surface and the adsorbate PTFE transfer film. Molecular dynamics simulations were used to calculate the interaction energy, in terms of different component of the transfer film and various Fe surfaces. The concentration profile of the adsorbate PTFE transfer film along the direction perpendicular to the Fe surface was discussed for the Fe/polymer interface systems. To reveal the bonding distance between the adsorbed transfer film and Fe surface, the radial distribution function of the inter-molecules was calculated for the Fe-F, Fe-C, and Fe-O atomic pairs. In addition, mean square displacement was employed to elucidate the dynamics of the polymer chains in the interface systems.

## Methodology

The Forcite module of the Materials Studio 7.0 was utilized to perform the molecular dynamics calculations. The ab initio COMPASS (condensed-phase optimized molecular potentials for atomistic simulation studies) forcefield was used for the molecular dynamics calculations. The PTFE molecule with ten repeat units was employed to build the PTFE layer, which is long enough to represent the PTFE (Zuo et al., 2015b). The Smart algorithm composed of the steepest descent, quasi-Newton, and adjusted basis set Newton-Raphson methods was adopted for the geometry optimization of interface models. During the geometry optimizations, the convergence condition of the energy change, force, stress, and displacement were less than 2 × 10^–5^ kcal/mol, 0.001 kcalmol^-1^Å^−1^, 0.001 GPa, 1 × 10^–5^ Å, respectively. The electrostatic and van der Waals non-bond interactions were described by the Ewald summation and atom based summation methods, respectively. The canonical NVT ensemble was employed for the molecular dynamics calculations. The Andersen algorithm was selected for the temperature control of dynamics simulations. The duration of the molecular dynamics was set as 10,000 ps.

**FIGURE 1:**
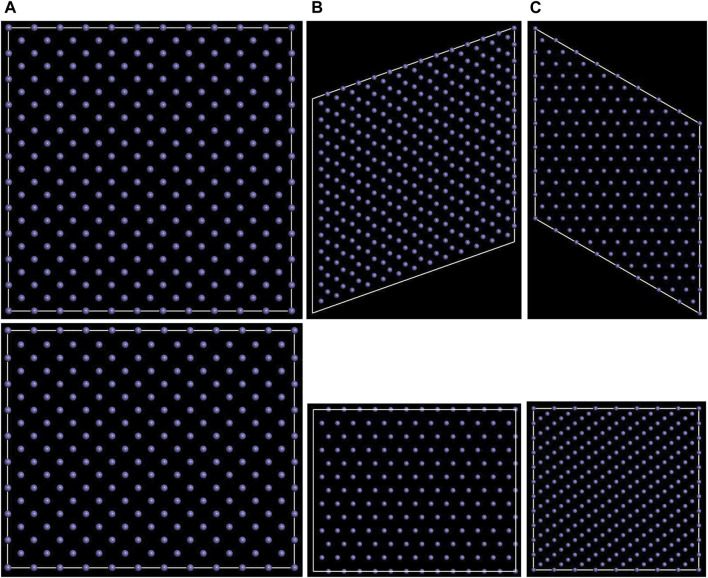
Top (above) and side (below) views for the **(A)** (100), **(B)** (110), and **(C)** (111) surface models of α-Fe.

Body-centred cubic Fe (α-Fe, JCPDS No. 6–0696) was used to build surface models. As shown in Figure 1, (100) (110), and (111) planes of α-Fe were considered to construct the Fe/polymer interface models. The layer numbers of 18, 12, and 33, and the super cells of 11 × 11, 13 × 13, and 8 × 8 were used to build the surface models of the (100), (110), and (111) planes, respectively. The length and width of the surface models both are larger than 3.1 nm, and the thicknesses are larger than 2.4 nm. The parameters of the (100), (110), and (111) super cell surface models are as follows: (100) surface, *a* × *b* × *c* = 31.53 Å × 31.53 Å × 26.05 Å, *α* = *β* = *γ* = 90^o^; (110) surface, *a* × *b* × *c* = 32.27 Å × 32.27 Å × 24.30 Å, *α* = *β* = 90^o^, *γ* = 70.53^o^; (111) surface, *a* × *b* × *c* = 32.43 Å × 32.43 Å × 27.43 Å, *α* = *β* = 90^o^, *γ* = 120^o^. Instead of the physical properties of metal crystal, the molecular dynamics simulation on the interface system mainly focused on the interaction between the adsorbed macromolecules and metal substrate, indicating that the thermal vibration of metal atoms can be ignored (Kornherr et al., 2003). Therefore, the atomic coordinate of the Fe atoms in the interface models was fixed. Based on the atomic trajectories obtained through the molecular dynamics, the interaction energy, concentration profile, radial distribution function, and mean square displacement were calculated to analyze the structure of the interface.

## Results and Discussion

### Adhesive Interaction Between PTFE and α-Fe Surfaces

#### Interaction Energy

The interaction energy (*E*_int_) between the substrate surface and adsorbate polymer can be calculated by (Nikkhah et al., 2015):

$$E_{\mathrm{int}}=\left(E_{\text{interface}}-E_{\text{Fe}}-E_{\text{polymer}}\right)/S$$ (1)

where *S* is the interfacial contact area, *E*_interface_, *E*_Fe_, and *E*_polymer_ are the total energies of the interfacial model, isolated Fe surface, and adsorbate polymer, respectively. The negative values of interaction energy correspond to the adhesive interaction between the substrate surface and adsorbate polymer. In addition, the non-bond interaction of interface is composed of van der Waals energy (*E*_vdW_) and electrostatic energy (*E*_Coul_):

$$E_{\mathrm{int}}=E_{Coul}+E_{vdW}$$ (2)

For the α-Fe/PTFE interface models, the coordinate axis perpendicular to the Fe surface is defined as z-axis. Due to the periodic boundary condition of the interface model, it is necessary to remove the interaction between the uppermost and the bottom atoms in the z-axis direction. This can be achieved by adding a thick vacuum layer above the adsorbate polymers. In this study, the vacuum slabs with the layer thickness of 200 Å were added in the α-Fe/PTFE interface models. The parameters of the (100)/PTFE, (110)/PTFE, and (111)/PTFE interface models are as follows: (100)/PTFE, *a* × *b* × *c* = 31.53 Å × 31.53 Å × 260.08 Å, *α* = *β* = *γ* = 90^o^; (110)/PTFE, *a* × *b* × *c* = 32.27 Å × 32.27 Å × 257.84 Å, *α* = *β* = 90^o^, *γ* = 70.53^o^; (111)/PTFE, *a* × *b* × *c* = 32.43 Å × 32.43 Å × 262.11 Å, *α* = *β* = 90^o^, *γ* = 120^o^.

The interaction energies of the (100)/PTFE, (110)/PTFE, and (111)/PTFE interface systems were −1.107, −1.276, and −1.042 kcal/molÅ^2^, respectively. These negative values demonstrated the adhesive interaction between the PTFE and α-Fe surfaces. The interaction energy of PTFE adhered on different Fe surfaces is ordered as (110) < (100) < (111). The interaction energy of (110)/PTFE interface system (−1.276 kcal/molÅ^2^) is lower than those of (100)/PTFE (−1.107 kcal/molÅ^2^) and (111)/PTFE (−1.042 kcal/molÅ^2^) interface systems, indicating that the (110) surface owns the strongest adhesive interaction with the adsorbate PTFE. In addition, the interaction energy of Fe/PTFE interface is totally composed of *E*_vdW_, indicating the adhesive interaction between the Fe surface and adsorbate PTFE is caused by van der Waals interaction. Therefore, among the three crystal planes of α-Fe, (110) surface has the strongest adhesion strength with the PTFE, and the adhesive interaction between the adsorbed PTFE and Fe surface is contributed by the van der Waals forces.

#### Concentration Distribution of Adsorbed PTFE Along the Z-Axis

**FIGURE 2:**
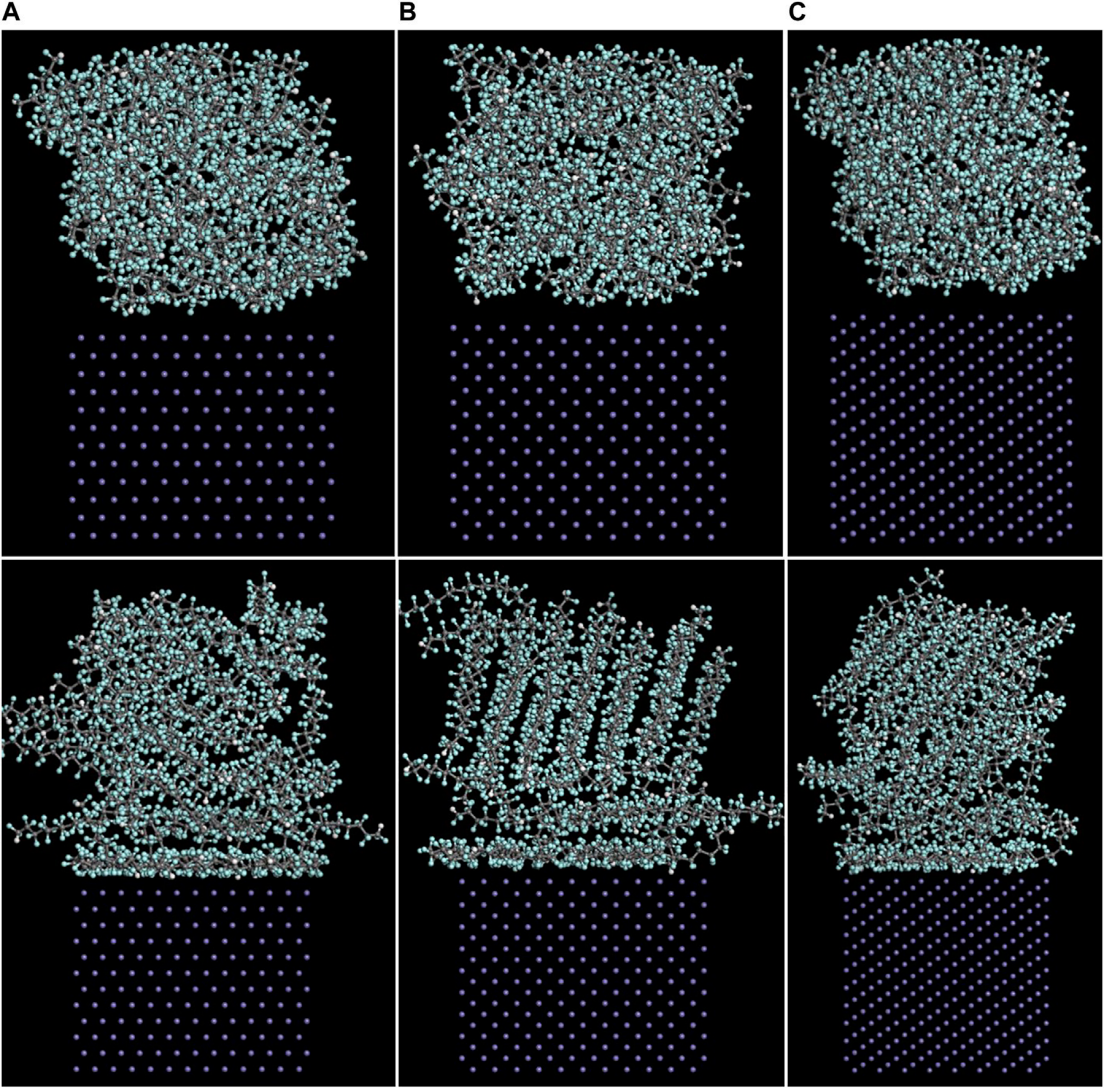
Side views of the **(A)** (110)/PTFE, **(B)** (100)/PTFE, and **(C)** (111)/PTFE interface models before (above) and after (below) the molecular dynamics.

The Fe/PTFE interface models before and after the molecular dynamics are shown in Figure 2. It can be clearly seen that a part of PTFE molecules in the interface is accumulated on the α-Fe surfaces after the molecular dynamics, which is caused by the adhesive interaction between the Fe surface and adsorbate PTFE.

**FIGURE 3:**
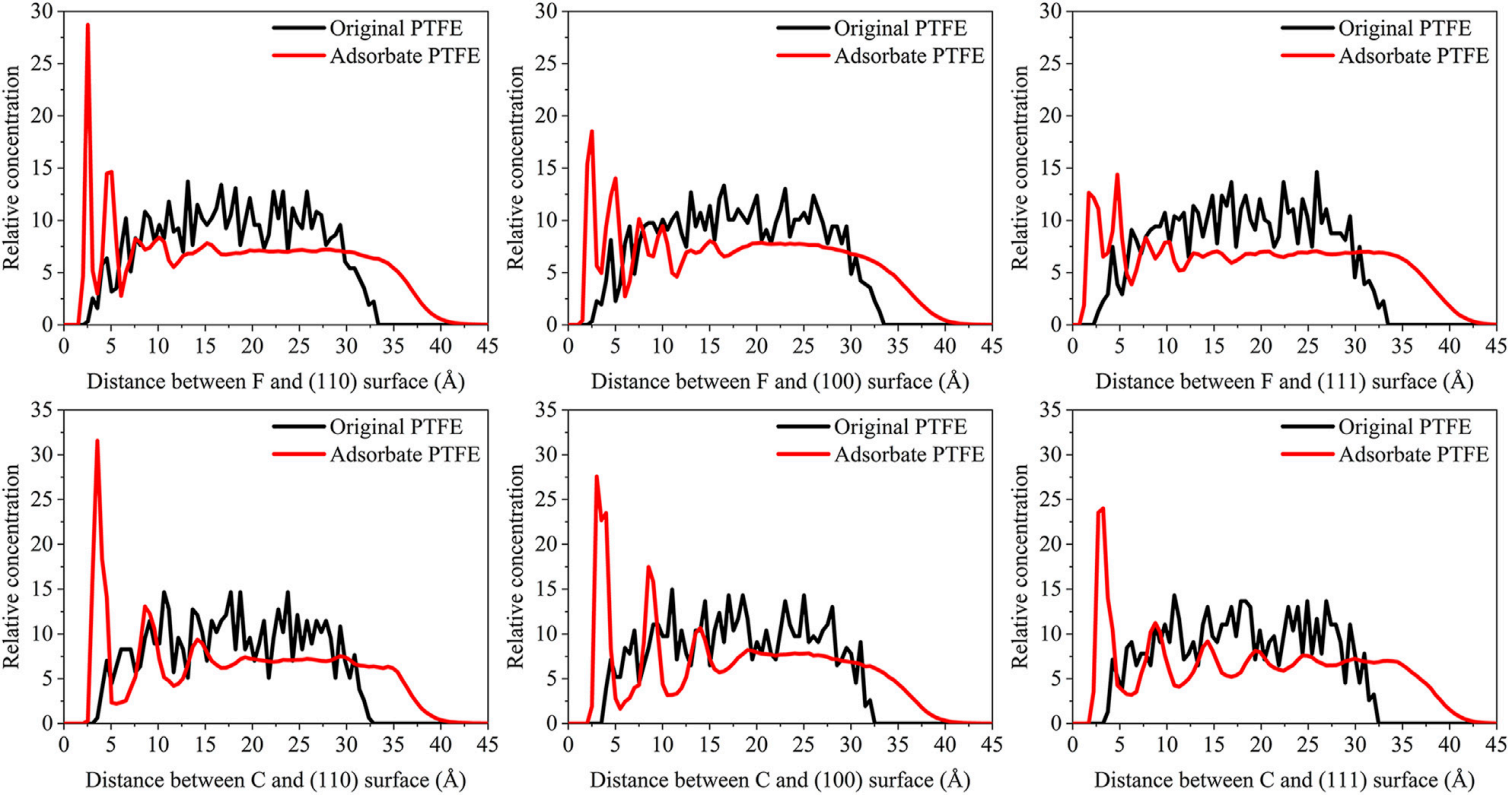
Concentration distribution of F and C atoms along z-axis for the (110)/PTFE (100)/PTFE, and (111)/PTFE interface systems.

The concentration distribution of adsorbate PTFE along the z-axis can be revealed intuitively by the concentration profile of the C and F atoms in the periodic interface system. The relative concentration (*R*) of the atoms can be calculated by:

$$R=\left(N_{\text{slab}}/V_{\text{slab}}\right)/\left(N_{\text{interface}}/V_{\text{interface}}\right)$$ (3)

where *N*_slab_ is the number of atoms in the slab perpendicular to the z-axis, *N*_interface_ is the number of atoms in the total interface system, *V*_slab_ and *V*_interface_ is the volume of the slab and total interface system, respectively. As shown in Figure 3, the abscissa represents the distance between the adsorbate atom and Fe surface, and the ordinate indicates the relative concentration of atoms.

For the original PTFE, F atoms are mainly located in the range of 2.5–33 Å from the α-Fe surface. However, after the adsorption of PTFE, F atoms are moved to the position of 2–40 Å. Especially, two high peaks of F atom are found around 2.5 and 5 Å, indicating that the PTFE is adsorbed and accumulated on the Fe surface. This agree well with the side views of the α-Fe/PTFE interface system before and after the molecular dynamics (Figure 2). The highest peak intensities of the F atom for the (110)/PTFE, (100)/PTFE, and (111)/PTFE interface systems were 28.7, 18.5, and 12.6, respectively, which is attributed to the difference in the adhesive interaction between the Fe surfaces and adsorbate PTFE. The stronger the adhesive interaction between the Fe surface and adsorbate PTFE, the higher is the peak intensity for the first peak of F atom.

Before the molecular dynamics, the C atoms can be found in the range of 3–32.5 Å from the α-Fe surface. After the adsorption of PTFE, the C atoms moved to the position of 2.5–40 Å. The high peaks locate at 3.55, 3.01, and 3.23 Å can be observed for the (110)/PTFE, (100)/PTFE, and (111)/PTFE interface systems, respectively, indicating the PTFE molecules are accumulated on the α-Fe surface. However, these distances (3.55, 3.01, and 3.23 Å) are larger than those of F atoms (2.5 Å), indicating that the Fe surface mainly interact with the F atoms of adsorbate PTFE. The highest peak intensities of the C atom for the (110)/PTFE, (100)/PTFE, and (111)/PTFE interface systems were 31.57, 27.58, and 24.01, respectively, which follows the sequence of (110)/PTFE > (100)/PTFE > (111)/PTFE. This result is consistent with the changing tendency of the concentration distribution of F atoms along z-axis, resulting from the difference in the adhesive interaction between the Fe surfaces and adsorbate PTFE.

#### Radial Distribution Function

The distance between the Fe atom and the atom of the adsorbate PTFE can be revealed by the radial distribution function (RDF) of inter-molecules. The radial distribution function g_AB_(r) can be calculated by (Luo and Jiang, 2010):

$${\text{g}}_{\text{AB}}\left(\text{r}\right)=\frac{1}{4\pi r^{2}\rho_{\text{AB}}}\frac{\sum_{t=1}^{S}\sum_{j=1}^{N_{\text{AB}}}\Delta N_{\text{AB}}\left(r\to r+\delta r\right)}{N_{\text{AB}}\times S}$$ (4)

where r is the distance from the reference atom, *ρ*_AB_ is the density of the interface system, *δ*r is the interval of distance, *N*_AB_ is the sum of the number for the atom A and atom B, Δ*N*_AB_ is the number of atoms within the distance of r ∼ r + δr from the reference atom, *S* is the number of step time.

**FIGURE 4:**
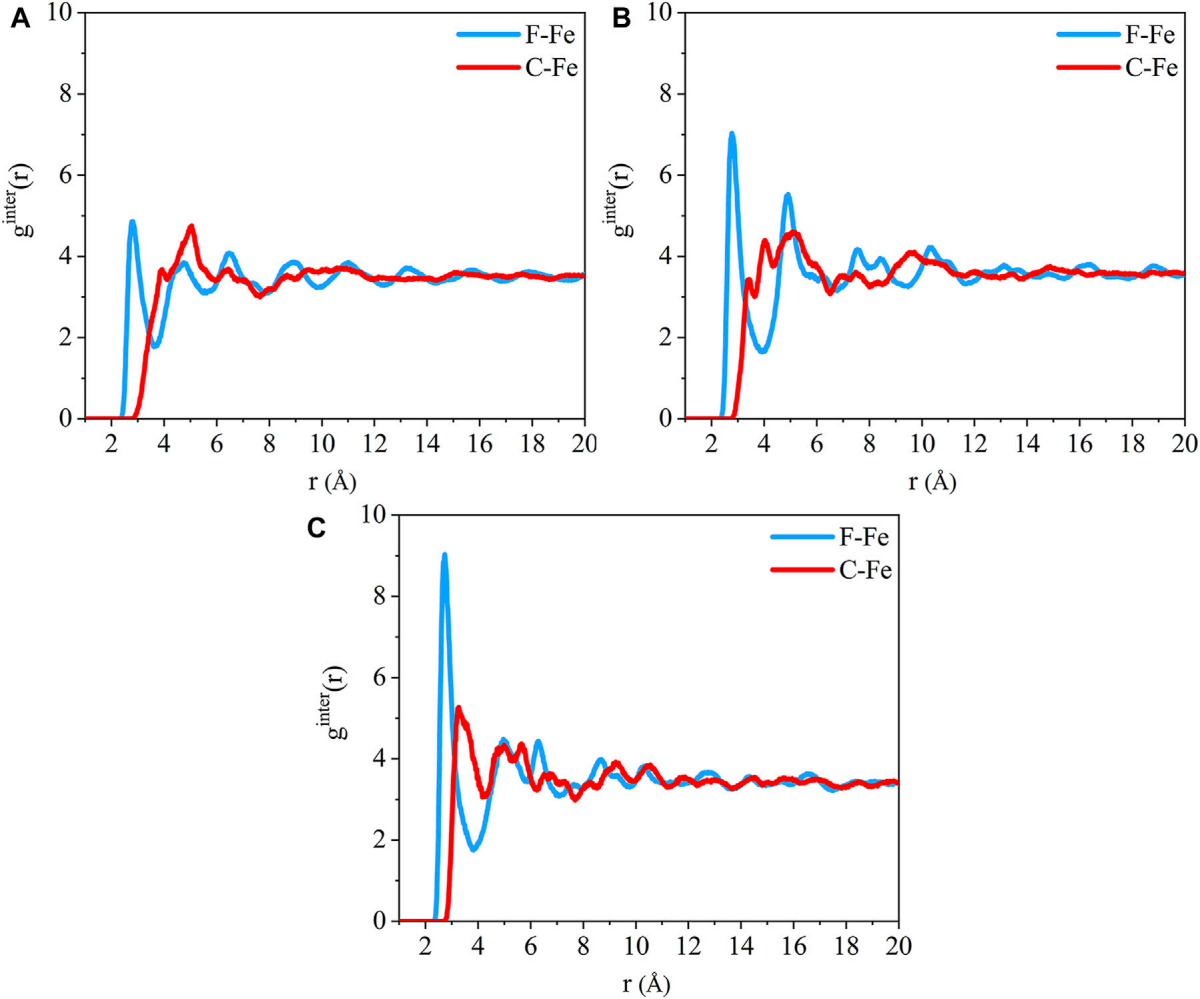
Radial distribution function of the F-Fe and C-Fe pairs for the **(A)** (110)/PTFE, **(B)** (100)/PTFE, and **(C)** (111)/PTFE interface systems.

The radial distribution function of the F-Fe and C-Fe pairs for the (110)/PTFE, (100)/PTFE, and (111)/PTFE interface systems are shown in Figure 4. Fe, F, and C represent the iron atom in the topmost layer of the α-Fe surface, the fluorine atom of the adsorbate PTFE, and the carbon atom of the adsorbate PTFE, respectively. The highest peak of the F-Fe pairs for the (100)/PTFE, (110)/PTFE, and (111)/PTFE interface systems locate at 2.77, 2.81, and 2.73 Å, respectively, which indicates the bonding distance between the Fe atom and the nearest F atom of the adsorbate PTFE. The first peak of the C-Fe pairs for the (100)/PTFE, (110)/PTFE, and (111)/PTFE interface systems locate at 3.45, 3.93, and 3.27 Å, respectively. These distances (3.45, 3.93, and 3.27 Å) are longer than those of F-Fe pairs (2.77, 2.81, and 2.73 Å), indicating that the Fe surface mainly interact with the F atoms. This agree well with the results of the concentration distribution of F and C atoms in the adsorbed PTFE along the z-axis.

The intensity for the first peak of the F-Fe pairs follows the sequence of (110)/PTFE < (100)/PTFE < (111)/PTFE, which is attributed to the difference in the atomic density of α-Fe surfaces. The atomic density of the α-Fe planes follows the sequence of (110) > (100) > (111) (Spencer et al., 2002). The intensity for the first peak of the F-Fe pairs increase with the decrease of the atomic density of α-Fe surfaces. As the close-packed plane of α-Fe, (110) surface owns the largest atomic density, leading to the (110)/PTFE interface system exhibits the lowest intensity for the first peak of F-Fe pairs. In addition, there is little difference in the position and intensity of the peaks for the radial distribution function of the C-Fe pairs, which is ascribed to the weak interaction between the carbon atoms of adsorbate PTFE and the topmost Fe layers.

#### Dynamics of Polymer Molecules

**FIGURE 5:**
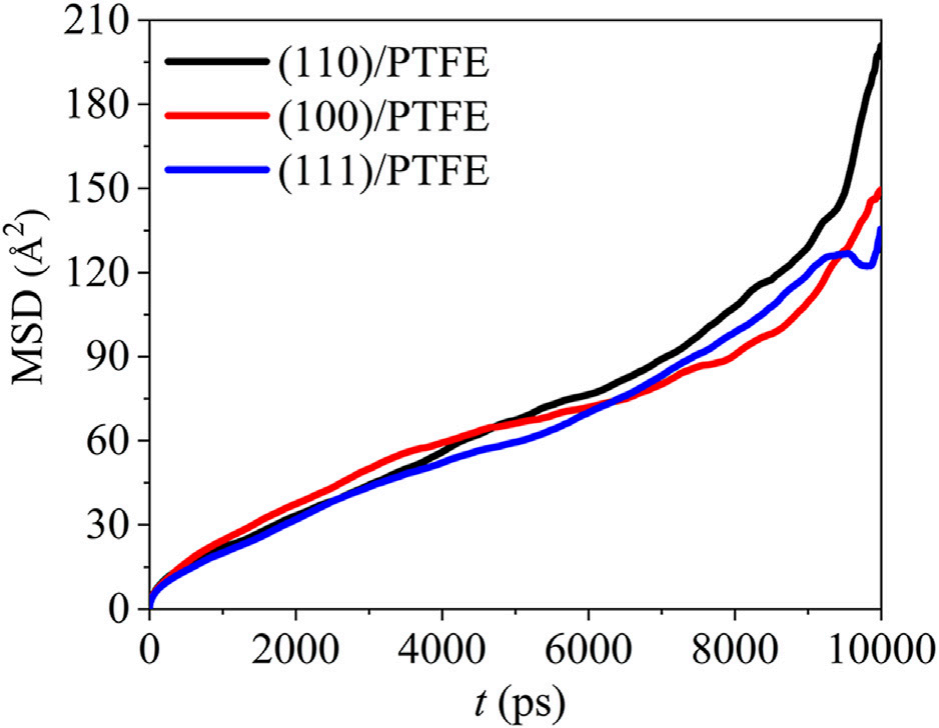
Mean square displacement of PTFE molecule in the (100)/PTFE (110)/PTFE, and (111)/PTFE interface systems.

Due to the mobility of polymer chains can be affected by the adhesion of Fe surfaces, polymers will exhibit various dynamic characteristics in different interface systems. The dynamics of the polymer chains can be revealed by the mean square displacement (MSD). Average value for the square of particle displacement relative to the initial position is defined as mean square displacement (MSD), which can be calculated by (Luo and Jiang, 2010):

$$MSD\left(\text{Δ}t\right)=\frac{1}{T-\Delta t}\int_{0}^{T-\Delta t}{\left[r\left(t-\Delta t\right)-r\left(t\right)\right]}^{2}\text{d}t=\langle {\left[r\left(t-\Delta t\right)-r\left(t\right)\right]}^{2}\rangle$$ (5)

where *T* is the total MD duration, r(*t*) and r(*t*−Δ*t*) are the position at the time of *t* and *t*−Δ*t*, respectively. The mean square displacement of the PTFE molecules in the (100)/PTFE, (110)/PTFE, and (111)/PTFE interface systems are shown in Figure 5. In the first 7,500 ps, the difference in the mobility of PTFE molecules in these three interface systems is very slight. Within the duration of 7,500–9,500 ps, the mobility of PTFE molecules follows the sequence of (100)/PTFE < (111)/PTFE < (110)/PTFE. But within the duration of 9,500–10,000 ps, the mobility of PTFE molecule in the (110)/PTFE interface system is higher than those of (100)/PTFE and (111)/PTFE interface systems, and the adsorbate PTFE on the (111) surface exhibits the lowest mobility.

### Adhesive Interaction Between PTFE-Based Polymer and α-Fe Surfaces.

#### Interaction Energy

Previous published studies demonstrated that the PTFE transfer film is composed of PTFE, hydroxyl substituted PTFE, carbonyl substituted PTFE, and chain broken PTFE (Krick et al., 2012; Zuo et al., 2014; Harris et al., 2015). In this study, the interaction between the component of PTFE transfer film (PTFE-OH, PTFE=O, and PTFE-S) and the Fe surfaces were also investigated by the molecular dynamics. The hydroxyl substituted PTFE, carbonyl substituted PTFE, and chain broken PTFE are represented as PTFE-OH, PTFE=O, and PTFE-S, respectively. The vacuum slabs with the layer thickness of 200 Å were added in the Fe/polymer interface models. The parameters of the Fe/polymer interface models are as follows: (100)/PTFE-S, *a* × *b* × *c* = 31.53 Å × 31.53 Å × 259.75 Å, *α* = *β* = *γ* = 90^o^; (100)/PTFE=O, *a* × *b* × *c* = 31.53 Å × 31.53 Å × 258.47 Å, *α* = *β* = *γ* = 90^o^; (100)/PTFE-OH, *a* × *b* × *c* = 31.53 Å × 31.53 Å × 260.05 Å, *α* = *β* = *γ* = 90^o^; (110)/PTFE-S, *a* × *b* × *c* = 32.27 Å × 32.27 Å × 257.44 Å, *α* = *β* = 90^o^, *γ* = 70.53^o^; (110)/PTFE=O, *a* × *b* × *c* = 32.27 Å × 32.27 Å × 256.86 Å, *α* = *β* = 90^o^, *γ* = 70.53^o^; (110)/PTFE-OH, *a* × *b* × *c* = 32.27 Å × 32.27 Å × 257.86 Å, *α* = *β* = 90^o^, *γ* = 70.53^o^; (111)/PTFE-S, *a* × *b* × *c* = 32.43 Å × 32.43 Å × 261.77 Å, *α* = *β* = 90^o^, *γ* = 120^o^; (111)/PTFE=O, *a* × *b* × *c* = 32.43 Å × 32.43 Å × 260.49 Å, *α* = *β* = 90^o^, *γ* = 120^o^; (111)/PTFE-OH, *a* × *b* × *c* = 32.43 Å × 32.43 Å × 262.07 Å, *α* = *β* = 90^o^, *γ* = 120^o^.

As detailed in Table 1, the interaction energy of the PTFE transfer film on three α-Fe surfaces all exhibited negative values, indicating the adhesive interaction between the polymer and α-Fe surfaces. The interaction energy of the PTFE-S, PTFE-OH, and PTFE=O molecules adhered on different Fe surfaces follows the sequence of (110) < (100) < (111), indicating that the (110) and (111) surfaces possess the strongest and weakest adhesive interaction with the PTFE-based polymer, respectively. Combined with the interaction energy results of PTFE, it demonstrates that the (110) surface owns the strongest adhesive interaction with the PTFE transfer film.

**TABLE 1:** The interaction energies of PTFE transfer film on α-Fe surfaces.

Surface	*E*_int_ (kcal/molÅ^2^)				Proportion of *E*_vdW_		
	PTFE-S	PTFE=O	PTFE-OH	PTFE	PTFE-S	PTFE=O	PTFE-OH
(110)	−1.332	−1.304	−1.307	−1.276	100%	100%	100%
(100)	−1.157	−1.132	−1.113	−1.107	100%	100%	100%
(111)	−1.064	−1.065	−1.077	−1.042	100%	100%	100%

Adhered on a same surface, the interaction energies of PTFE-S, PTFE-OH, and PTFE=O molecules are always lower than that of PTFE molecule, indicating that the hydroxyl substitution, carbonyl substitution, and chain scission reactions of PTFE increase the adhesive interaction of PTFE during the generation of PTFE transfer film. For instance, the interaction energies of PTFE-S, PTFE=O, and PTFE-OH (−1.332, −1.304, −1.307 kcal/mol Å^2^) adhered on the (110) surface are all slightly lower than that of PTFE (−1.276 kcal/mol Å^2^). The smallest value of the interaction energy of PTFE-S on (110) surface (−1.332 kcal/mol Å^2^) demonstrating the strongest adhesive interaction between the PTFE-S and (110) surface. There is only a marginal difference in the interaction energies of PTFE=O (−1.304 kcal/mol Å^2^) and PTFE-OH (−1.307 kcal/mol Å^2^) adhered on the (110) surface, but they are slightly larger than that of PTFE-S (−1.332 kcal/mol Å^2^). Moreover, the interaction energy of Fe/polymer interface systems is composed entirely of *E*_vdW_ (100%), which is accordant with the Fe/PTFE interface systems. This indicates that the adhesive interaction between the Fe surface and PTFE transfer film are dominated by the van der Waals force. Compared with the original PTFE molecule, PTFE-OH and PTFE=O molecules exhibit stronger adhesive interaction with α-Fe, which could provide guidance in enhancing the adhesion strength of PTFE transfer film on the iron surface. We suppose that the introduction of carbonyl and hydroxyl into the PTFE molecule before friction might be conducive to the formation of PTFE transfer film, thereby improving the lubricating performance of PTFE. And the generation of carbonyl and hydroxyl can be achieved through the surface modification of PTFE.

#### Concentration Distribution of Adsorbate Polymer Along the Z-Axis

**FIGURE 6:**
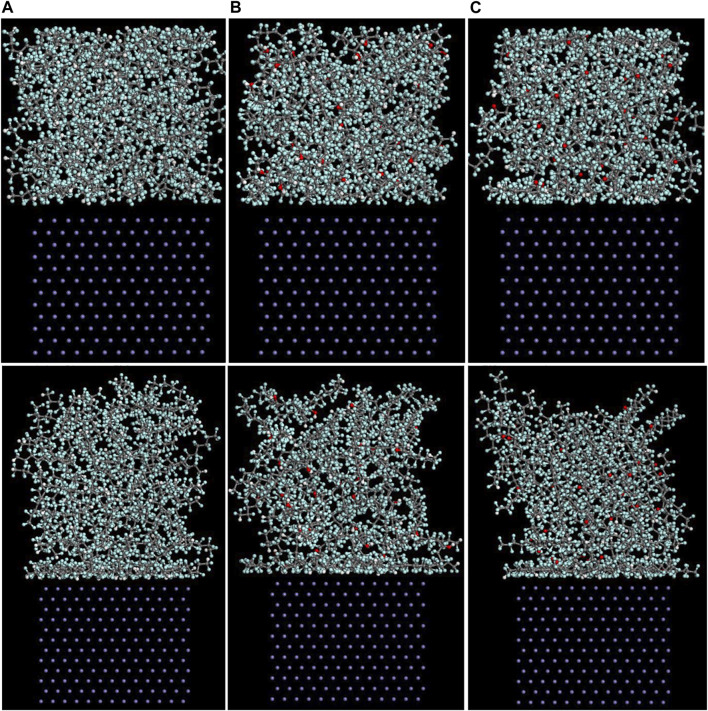
Side views of the **(A)** (110)/PTFE-S, **(B)** (110)/PTFE-OH, and **(C)** (111)/PTFE = O interface systems before (above) and after (below) the molecular dynamics.

**FIGURE 7:**
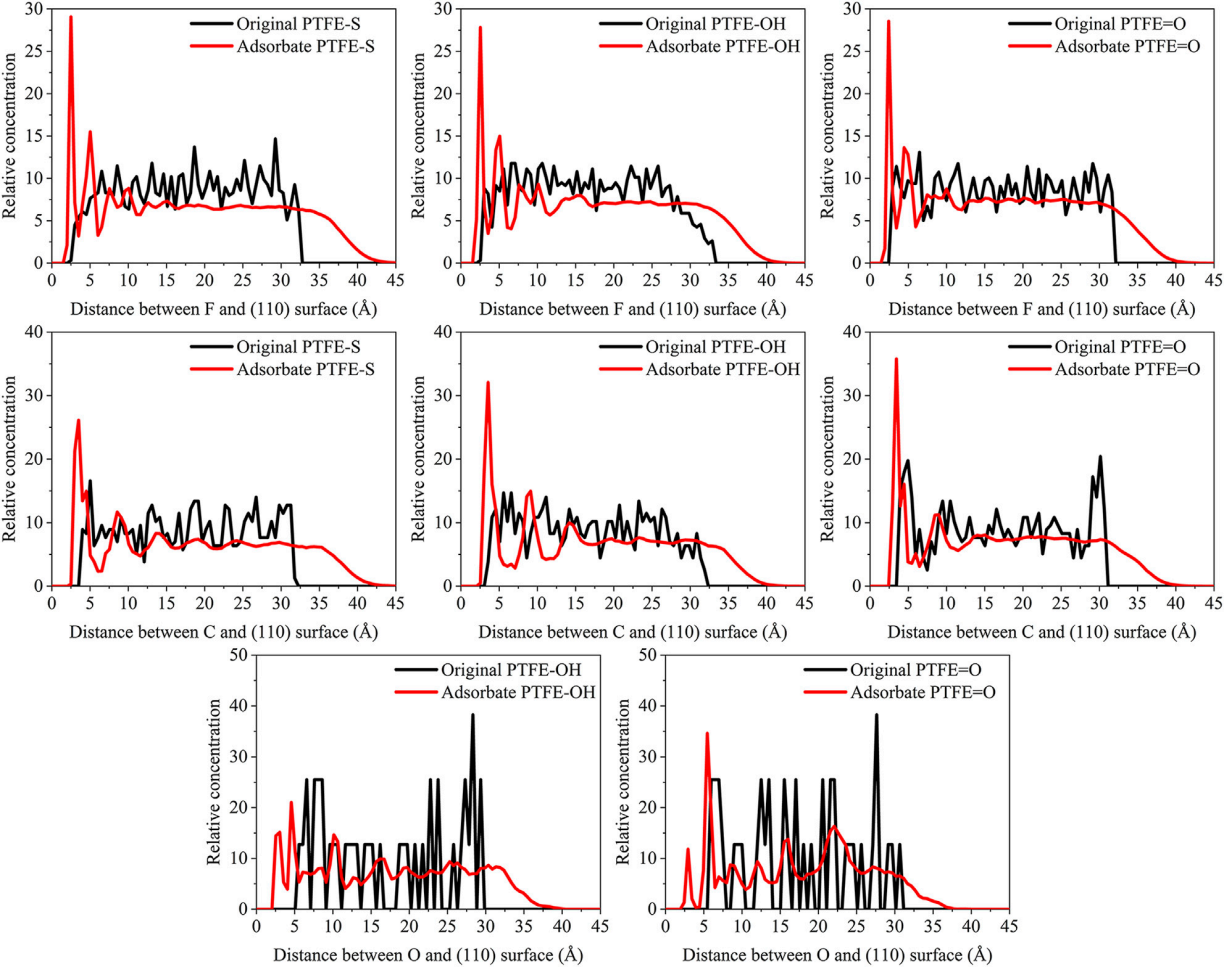
Concentration distribution of F, C, and O atoms along z-axis for the (110)/PTFE-S, (110)/PTFE-OH, and (110)/PTFE = O interface systems.

As mentioned above, among the (100), (110), and (111) surfaces of α-Fe, the (110) surface has the strongest adhesive interaction with the PTFE-S, PTFE=O, and PTFE-OH molecules. Therefore, the (110)/polymer interface models and the concentration distribution of adsorbed PTFE-S, PTFE-OH, and PTFE=O on the (110) surface along the z-axis before and after molecular dynamics are illustrated in Figures 6, 7, respectively. As shown in Figure 6, before the molecular dynamics, the PTFE-OH, PTFE=O, and PTFE-S molecules do not aggregate on the (110) surface of α-Fe. But after the molecular dynamics, it can be seen intuitively that PTFE-OH, PTFE=O, and PTFE-S molecules are accumulated and piled up on the (110) surface of α-Fe. This phenomenon is caused by the adhesive interaction of the PTFE-OH, PTFE=O, and PTFE-S molecules on the (110) surface.

As shown in Figure 7, for the original polymers, F atoms are located at the position of 2.5–33 Å from the Fe surface. But the F atoms are moved toward the Fe surface obviously after the molecular dynamics, corresponding to the high peaks locate around 2 and 5 Å from the Fe surface. This indicates that the PTFE-OH, PTFE=O, and PTFE-S molecules are transferred and accumulated on the (110) surface. Due to the deviation of *E*_int_ for the (110)/PTFE-OH, (110)/PTFE=O, and (110)/PTFE-S interface systems are less than 3%, there is little difference in the intensity of the highest peaks. This agree well with the side views of the (110)/PTFE-OH, (110)/PTFE=O, and (110)/PTFE-S interface systems before and after the molecular dynamics (Figure 6).

Before the molecular dynamics, C atoms can be found in the range of 3–32 Å from the (110) surface. After the molecular dynamics of the interface systems, C atoms are moved to the positions of 2.5–42 Å. The high peaks locate at 3.51, 3.55, and 3.45 Å can be observed for the (110)/PTFE-S, (110)/PTFE-OH, and (110)/PTFE=O interface systems, respectively, demonstrating that the PTFE-S, PTFE-OH, and PTFE=O molecules are accumulated on the (110) surface. The position of the high peak of the C atoms (3.51, 3.55, and 3.45 Å) are larger than those of F atoms (2.50, 2.54, 2.44 Å), indicating that the Fe surface mainly interact with the F atoms of adsorbate PTFE.

For the (110)/PTFE=O and (110)/PTFE-OH interface systems, O atoms are the characteristic atoms of PTFE=O and PTFE-OH molecules. Thus, the concentration profile of O atoms for the (110)/PTFE-OH and (110)/PTFE=O interface systems can be seen in Figure 7. Before the molecular dynamics, O atoms are mainly distributed within the distance range of 5–30 Å from the α-Fe surface. After the molecular dynamics, the position of the concentration peak moves toward the Fe surface, especially two new peaks appeared around 3 and 5 Å. This demonstrates the accumulation of PTFE=O and PTFE-OH molecules on the (110) surface, agreeing well with the concentration distribution of F and C atoms.

#### Radial Distribution Function

**FIGURE 8:**
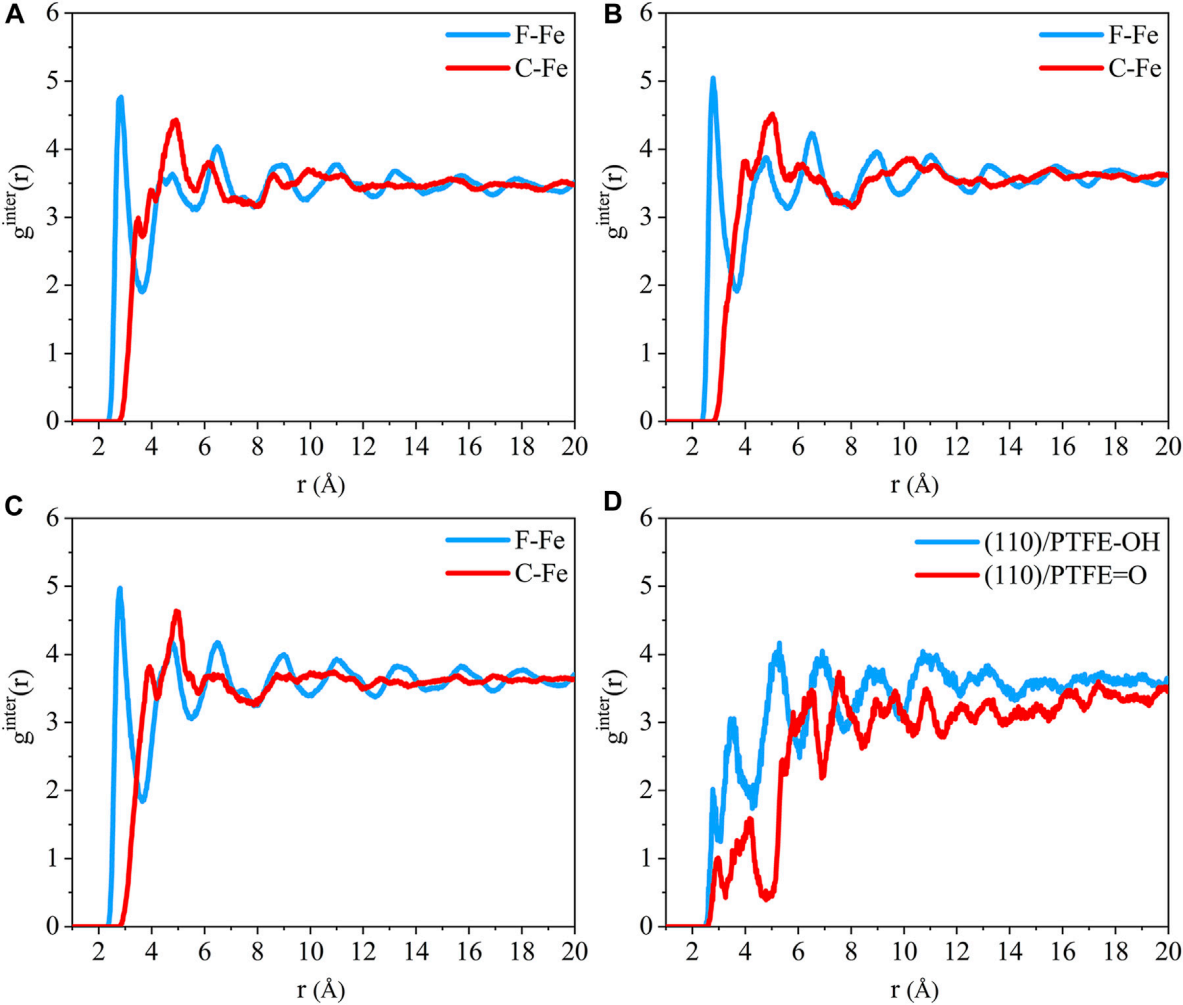
Radial distribution function of the F-Fe and C-Fe pairs for the **(A)** (110)/PTFE-S, **(B)** (110)/PTFE-OH, and **(C)** (110)/PTFE = O interface systems. Radial distribution function of the **(D)** O-Fe pairs for the (110)/PTFE-OH and (110)/PTFE = O interface systems.

The radial distribution of the F-Fe and C-Fe pairs for the (110)/PTFE-S, (110)/PTFE-OH, and (110)/PTFE=O interface systems are shown in Figures 8A–C. Fe, F, and C represent the iron atom in the topmost layer of the (110) surface, the fluorine atom of the adsorbate PTFE, and the carbon atom of the adsorbate PTFE, respectively. The highest peak of the F-Fe pairs for the (110)/PTFE-S, (110)/PTFE-OH, and (110)/PTFE=O interface systems locate at 2.85, 2.79, and 2.81 Å, respectively, which indicates the bonding distance between the Fe atom and the nearest F atom of adsorbate macromolecules. There is only a marginal difference in the peak intensity of F-Fe pairs between these three different interface systems.

The first peak of the C-Fe pairs for the (110)/PTFE-S, (110)/PTFE-OH, and (110)/PTFE=O interface systems locate at 3.49, 4.01, and 3.93 Å, respectively. These distances (3.49, 4.01, and 3.93 Å) are longer than those of F-Fe pairs (2.85, 2.79, and 2.81 Å), demonstrating that the (110) surface mainly interact with the F atom of polymers. This agree well with the results of the concentration distribution of F and C atoms in the adsorbed PTFE along the z-axis. There is little difference in the peak intensity of C-Fe pairs between these three different interface systems.

The radial distribution of the O-Fe pairs for the (110)/PTFE-OH and (110)/PTFE=O interface systems are shown in Figure 8D. The first peak of the O-Fe pairs can reveal the bonding distance between the Fe atom and the nearest O atom of the adsorbate macromolecules. The first peak of the (110)/PTFE-OH and (110)/PTFE=O interface systems locate at 2.77 and 2.91 Å, respectively. The peak intensity for the first peak of the (110)/PTFE-OH interface system is smaller than that of the (110)/PTFE=O interface system, indicating that more Fe-O bonds were formed between the Fe surface and the O atoms in the PTFE-OH.

#### Dynamics of Polymer Molecules

**FIGURE 9:**
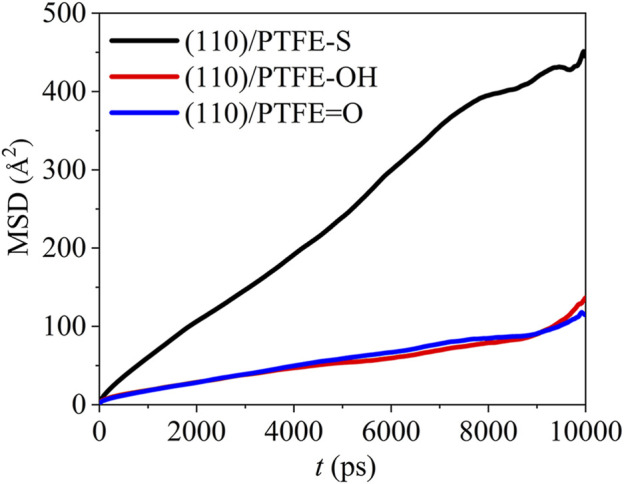
Mean square displacement of polymer molecules in the (110)/PTFE = O, (110)/PTFE-OH, and (110)/PTFE-S interface systems.

The mean square displacement of the PTFE-based polymers in the (110)/PTFE=O, (110)/PTFE-OH, and (110)/PTFE-S interface systems can be seen in Figure 9. There is only a marginal difference in the mobility of PTFE=O and PTFE-OH molecules in the (110)/PTFE=O and (110)/PTFE-OH interface systems. But the mobility of PTFE-S molecule is far higher than those of PTFE=O and PTFE-OH molecules. This is due to the fact that the chain broken decreases the chain length of PTFE molecule, leading to the increase in the mobility of PTFE-S molecule.

### Adhesive Interaction Under Various Temperatures

#### Interaction Energy

As mentioned above, among the (100), (110), (111) surfaces of α-Fe, the (110) surface has the strongest adhesive interaction with the PTFE transfer film. Thus, the adhesive interaction between the (110) surface and PTFE under various temperatures were analyzed by molecular dynamics. The interaction energy of the (110)/PTFE interface system under the temperatures of 25, 100, 200, and 300°C were −1.276, −1.303, −1.297, and −1.259 kcal/molÅ^2^, respectively. These negative values indicate the adhesive interaction between the PTFE and (110) surfaces under different temperatures. The interaction energy at 100 and 200°C exhibit lower values than those of 25 and 300°C, but there is little difference in the interaction energy among different temperature (<3.5%). The interaction energy under different temperatures is composed entirely of *E*_vdW_, indicating that the adhesive interaction between the (110) surface and PTFE is always contributed by the van der Waals forces.

#### Concentration Distribution of Adsorbed PTFE Along the Z-Axis

**FIGURE 10:**
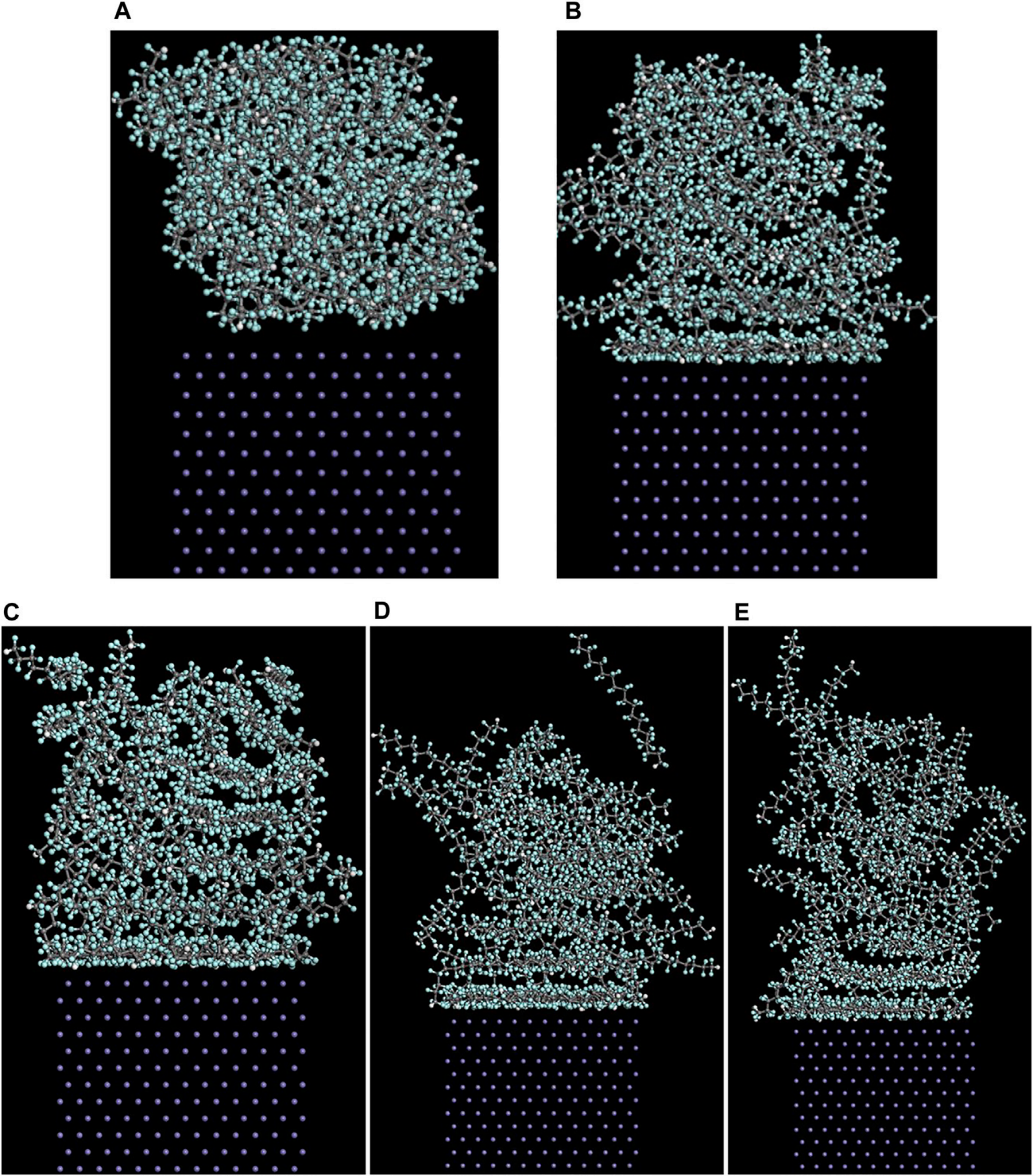
Side views of the (110)/PTFE interface models **(A)** before and after the molecular dynamics of different temperatures: **(B)** 25°C **(C)** 100°C, **(D)** 200°C, and **(E)** 300°C.

The (110)/PTFE interface models after the molecular dynamics of different temperatures are shown in Figure 10. A part of PTFE molecules in the interface is accumulated on the (110) surface of α-Fe, which is caused by the adhesive interaction between the PTFE and (110) surface. In addition, with the increase of temperature, the PTFE molecules moved a farther distance towards the direction of vacuum layer, and this will be quantitatively characterized by the concentration profile along the z-axis.

**FIGURE 11:**
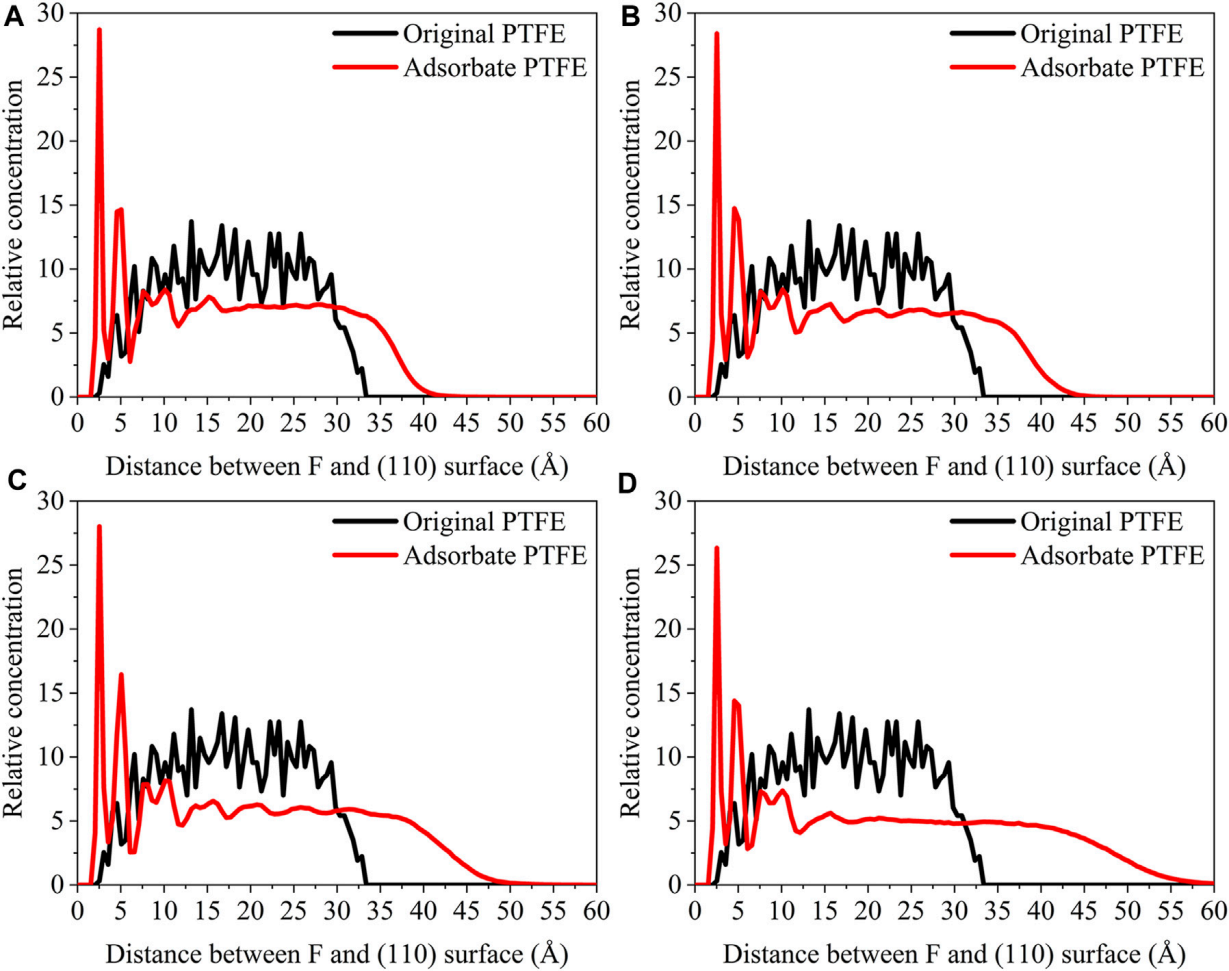
Concentration distribution of F atoms along the z-axis for the (110)/PTFE interface system under different temperatures: **(A)** 25°C, **(B)** 100°C, **(C)** 200°C, and **(D)** 300°C.

**FIGURE 12:**
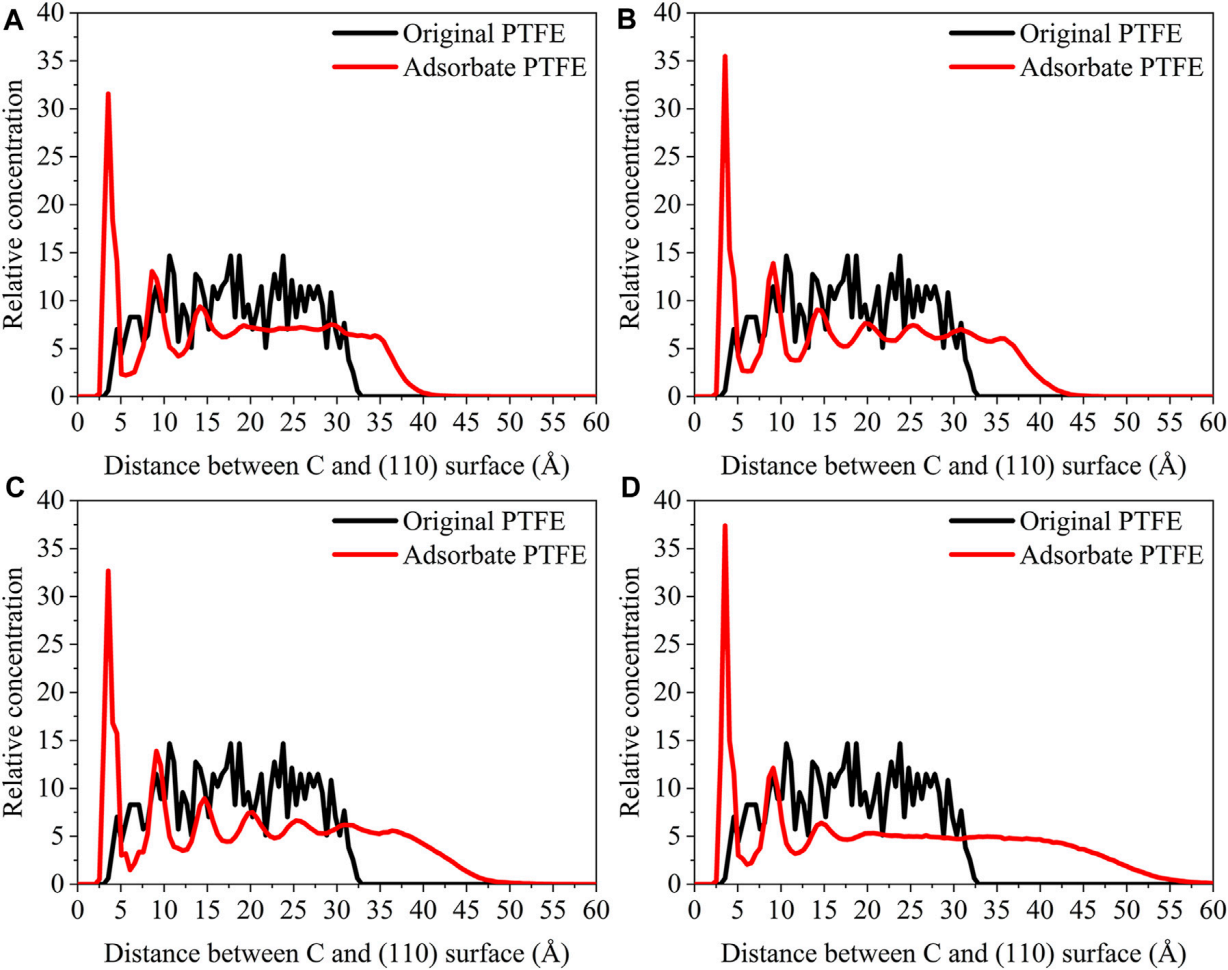
Concentration distribution of C atoms along the z-axis for the (110)/PTFE interface system under different temperatures: **(A)** 25°C, **(B)** 100°C, **(C)** 200°C, and **(D)** 300°C.

The concentration distribution of F atoms along the z-axis for the (110)/PTFE interface system under different temperatures can be seen in Figure 11. Before the molecular dynamics, F atoms can be found in the range of 2.5–33 Å from the (110) surface. After the molecular dynamics, F atoms of (110)/PTFE interface system under the temperatures of 25, 100, 200, and 300 C were moved to the locations of 1.5–40, 1.5–42, 1.5–47, and 1.5–55Å, respectively. The difference in the range of abscissa is consistent with Figure10, which is attributed to the increase of the molecule chain mobility with the increasing of temperature (Figure 14). In addition, two high peaks of F atom can be observed around 2.5 and 5 Å for the (110)/PTFE interface system under different temperatures, indicating that the PTFE is adsorbed and aggregated on the Fe surface. This agree well with the side views of the (110)/PTFE interface system under different temperatures (Figure 12).

As shown in Figure 12, for the original PTFE, C atoms can be found in the range of 3–32.5 Å from the (110) surface. After the adsorption of PTFE, C atoms of (110)/PTFE interface system under the temperatures of 25, 100, 200, and 300 C were mainly distributed in the range of 2.5–40, 2.5–42, 2.5–47, and 2.5–55Å, respectively. This is attributed to the increase of the mobility of polymer chain with the increasing of temperature (Figure 14). In addition, two high peaks can be observed around 3.5 and 9 Å for the (110)/PTFE interface system after the molecular dynamics, indicating that the PTFE is adsorbed and aggregated on the (110) surface. This agree well with the side views of the (110)/PTFE interface system after the molecular dynamics (Figure 12). These two distances (3.5 and 9 Å) are larger than those of F atoms (2.5 and 5 Å), indicating the Fe surface mainly interact with the F atoms of adsorbate PTFE.

#### Radial Distribution Function

**FIGURE 13:**
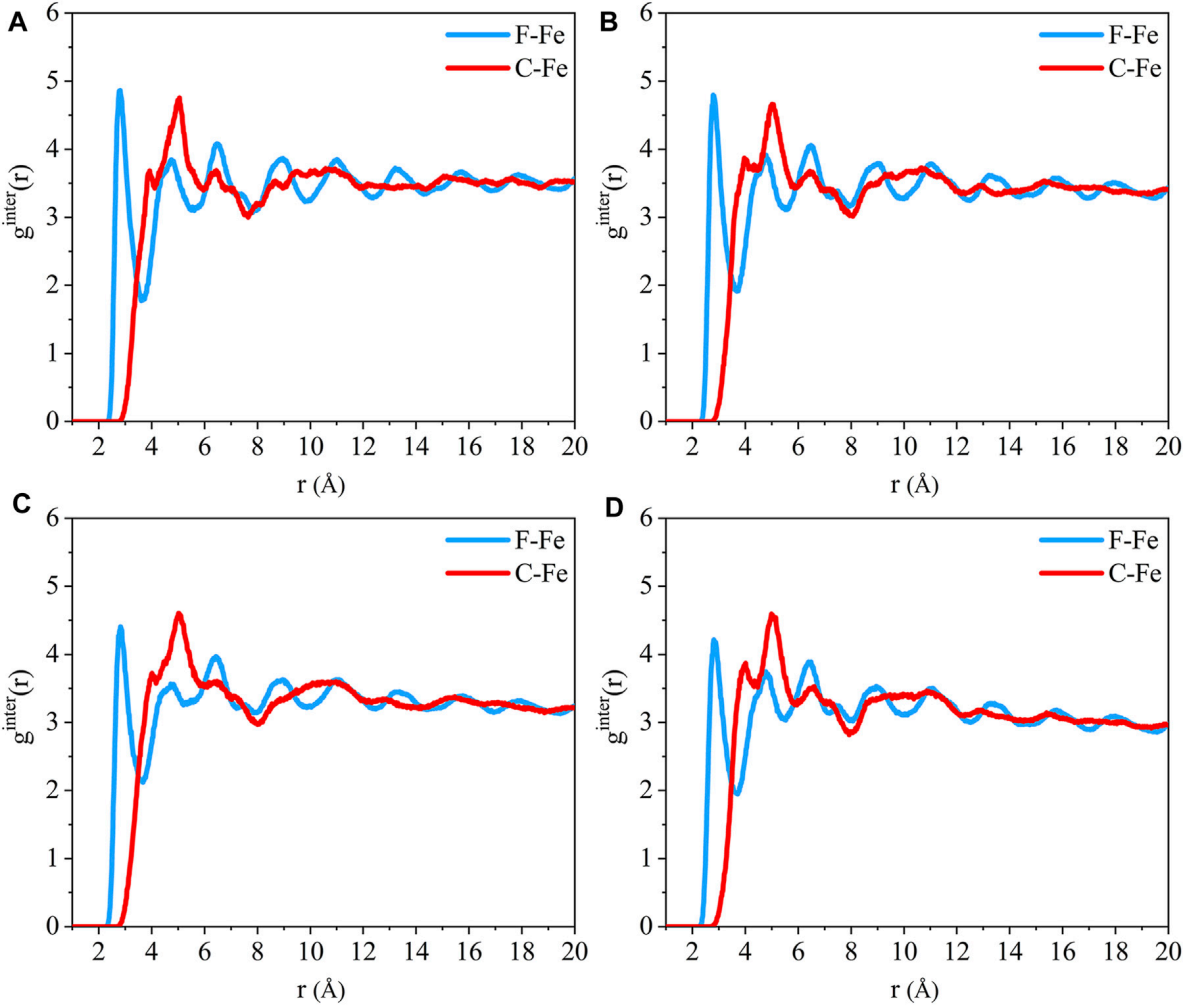
Radial distribution function of the F-Fe and C-Fe pairs for the (110)/PTFE interface system under various temperatures: **(A)** 25°C, **(B)** 100°C, **(C)** 200°C, and **(D)** 300°C.

The radial distribution function of the F-Fe and C-Fe pairs for the (110)/PTFE interface system under different temperatures are shown in Figure 13. Fe, F, and C represent the iron atom in the topmost layer of the (110) surface, the fluorine atom of the adsorbate PTFE, and the carbon atom of the adsorbate PTFE, respectively. The first peak of the F-Fe pairs for the (110)/PTFE interface system locates at 2.81 Å, which indicates the bonding distance between the Fe atom and the nearest F atom of the adsorbate PTFE. The first peak of the C-Fe pairs for the (110)/PTFE interface system locate at 4 Å, which is longer than that of F-Fe pairs (2.81 Å). This indicates that the Fe surface mainly interact with the F atoms, corresponding to the concentration profile of F and C atoms along the z-axis.

#### Dynamics of PTFE Molecules

**FIGURE 14:**
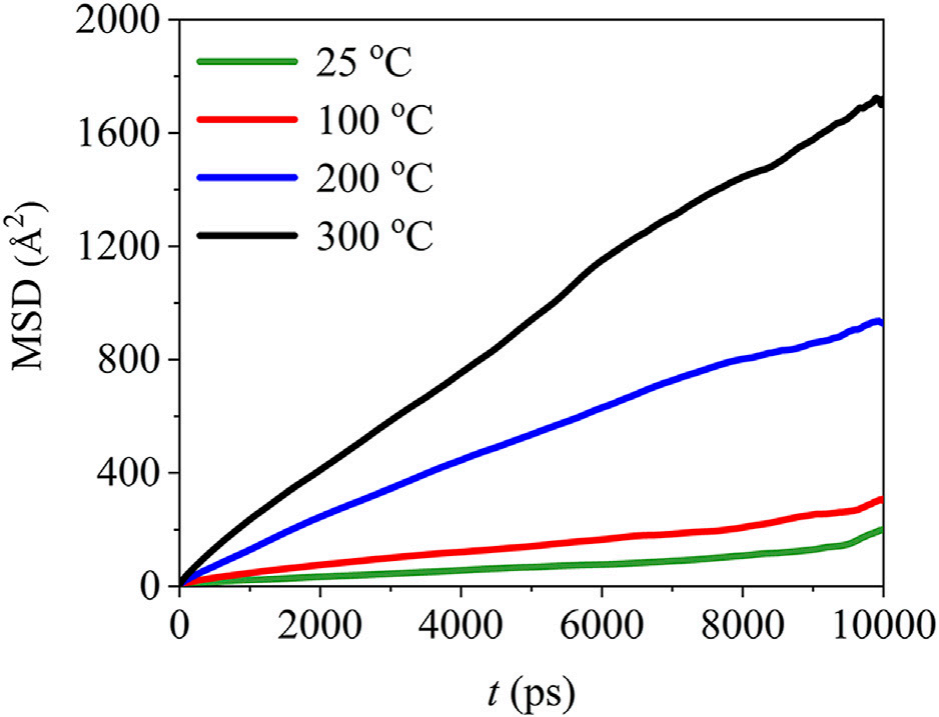
Mean square displacement of PTFE molecules in the (110)/PTFE interface system under different temperatures.

The mean square displacement of PTFE molecules in the (110)/PTFE interface system under different temperatures are shown in Figure 14. Temperature exhibits significant influence on the mean square displacement of PTFE molecules. The mobility of PTFE molecules increases remarkably with the rising of temperature. Especially, the mean square displacement of the PTFE molecules at 200 and 300 C are far larger than those of 25 and 100°C, indicating that the PTFE molecules own higher mobility under high temperatures.

## Conclusion

In this study, the interaction between the PTFE transfer film and iron surface was investigated by the molecular dynamics calculations. The interaction energy between the iron surface and PTFE transfer film was negative, demonstrating the adhesive interfacial interaction. This leads to the accumulation of PTFE transfer film on the Fe surface. Among the (100), (110), and (111) surfaces of α-Fe, (110) surface owns the strongest adhesive interaction with the PTFE transfer film. Compared with the original PTFE molecule, PTFE-S, PTFE-OH, and PTFE=O molecules exhibit stronger adhesive interaction with α-Fe.

The adhesive interaction between the adsorbate PTFE transfer film and Fe surface is contributed by the van der Waals energy, which is originated from the Fe surface and the F atoms of the adsorbate PTFE transfer film. The bonding distances between the Fe atom and F atom of the adsorbate polymer for the (100)/PTFE, (110)/PTFE, (111)/PTFE, (110)/PTFE-S, (110)/PTFE-OH, and (110)/PTFE=O interface systems are 2.77, 2.81, 2.73, 2.85, 2.79, and 2.81 Å, respectively. The bonding distances between the Fe atom and O atom of the adsorbate polymer for the (110)/PTFE-OH and (110)/PTFE=O interface systems are 2.77 and 2.91 Å, respectively. In addition, the chain broken of PTFE molecule and the increase of temperature remarkably increase the mobility of polymer chains.

## Data Availability

The raw data supporting the conclusions of this article will be made available by the authors, without undue reservation.

## References

[B1] Bahadur S. (2000). The Development of Transfer Layers and Their Role in Polymer Tribology. Wear 245, 92–99. 10.1016/S0043-1648(00)00469-5

[B2] Chiu P. Y. Barry P. R. Perry S. S. Sawyer W. G. Phillpot S. R. Sinnott S. B. (2011). Influence of the Molecular Level Structure of Polyethylene and Polytetrafluoroethylene on Their Tribological Response. Tribol. Lett. 42, 193–201. 10.1007/s11249-011-9763-0

[B3] Ewen J. P. Heyes D. M. Dini D. (2018). Advances in Nonequilibrium Molecular Dynamics Simulations of Lubricants and Additives. Friction 6, 349–386. 10.1007/s40544-018-0207-9

[B4] Friedrich K. Zhang Z. Schlarb A. (2005). Effects of Various Fillers on the Sliding Wear of Polymer Composites. Composites Sci. Technology 65, 2329–2343. 10.1016/j.compscitech.2005.05.028

[B5] Harris K. L. Pitenis A. A. Sawyer W. G. Krick B. A. Blackman G. S. Kasprzak D. J. Junk C. P. (2015). PTFE Tribology and the Role of Mechanochemistry in the Development of Protective Surface Films. Macromolecules 48, 3739–3745. 10.1021/acs.macromol.5b00452

[B6] Jang I. Burris D. L. Dickrell P. L. Barry P. R. Santos C. Perry S. S. Phillpot S. R. Sinnott S. B. Sawyer W. G. (2007). Sliding Orientation Effects on the Tribological Properties of Polytetrafluoroethylene. J. Appl. Phys. 102, 123509. 10.1063/1.2821743

[B7] Javan Nikkhah S. Moghbeli M. R. Hashemianzadeh S. M. (2015). Investigation of the Interface between Polyethylene and Functionalized Graphene: a Computer Simulation Study. Curr. Appl. Phys. 15, 1188–1199. 10.1016/j.cap.2015.07.007

[B8] Johnston J. P. Koo B. Subramanian N. Chattopadhyay A. (2017). Modeling the Molecular Structure of the Carbon Fiber/polymer Interphase for Multiscale Analysis of Composites. Composites B: Eng. 111, 27–36. 10.1016/j.compositesb.2016.12.008

[B9] Kornherr A. Hansal S. Hansal W. E. G. Besenhard J. O. Kronberger H. Nauer G. E. Zifferer G. (2003). Molecular Dynamics Simulations of the Adsorption of Industrial Relevant Silane Molecules at a Zinc Oxide Surface. J. Chem. Phys. 119, 9719–9728. 10.1063/1.1615491

[B10] Krick B. A. Ewin J. J. Blackman G. S. Junk C. P. Gregory Sawyer W. (2012). Environmental Dependence of Ultra-low Wear Behavior of Polytetrafluoroethylene (PTFE) and Alumina Composites Suggests Tribochemical Mechanisms. Tribology Int. 51, 42–46. 10.1016/j.triboint.2012.02.015

[B11] Liu F. Hu N. Ning H. Liu Y. Li Y. Wu L. (2015). Molecular Dynamics Simulation on Interfacial Mechanical Properties of Polymer Nanocomposites with Wrinkled Graphene. Comput. Mater. Sci. 108, 160–167. 10.1016/j.commatsci.2015.06.023

[B12] Luo Z. Jiang J. (2010). Molecular Dynamics and Dissipative Particle Dynamics Simulations for the Miscibility of Poly(ethylene Oxide)/poly(vinyl Chloride) Blends. Polymer 51, 291–299. 10.1016/j.polymer.2009.11.024

[B13] Moon J. Yang S. Cho M. (2017). Interfacial Strengthening between Graphene and Polymer through Stone-Thrower-wales Defects: Ab Initio and Molecular Dynamics Simulations. Carbon 118, 66–77. 10.1016/j.carbon.2017.03.021

[B14] Onodera T. Kawasaki K. Nakakawaji T. Higuchi Y. Ozawa N. Kurihara K. Kubo M. (2014). Effect of Tribochemical Reaction on Transfer-Film Formation by Poly(tetrafluoroethylene). J. Phys. Chem. C 118, 11820–11826. 10.1021/jp503331e

[B15] Onodera T. Nunoshige J. Kawasaki K. Adachi K. Kurihara K. Kubo M. (2017). Structure and Function of Transfer Film Formed from PTFE/PEEK Polymer Blend. J. Phys. Chem. C 121, 14589–14596. 10.1021/acs.jpcc.7b02860

[B16] Onodera T. Park M. Souma K. Ozawa N. Kubo M. (2013). Transfer-film Formation Mechanism of Polytetrafluoroethylene: A Computational Chemistry Approach. J. Phys. Chem. C 117, 10464–10472. 10.1021/jp400515j

[B17] Pan D. Fan B. Qi X. Yang Y. Hao X. (2019). Investigation of PTFE Tribological Properties Using Molecular Dynamics Simulation. Tribol. Lett. 67, 28. 10.1007/s11249-019-1141-3

[B18] Spencer M. J. S. Hung A. Snook I. K. Yarovsky I. (2002). Density Functional Theory Study of the Relaxation and Energy of Iron Surfaces. Surf. Sci. 513, 389–398. 10.1016/S0039-6028(02)01809-5

[B19] Unal H. Mimaroglu A. Kadıoglu U. Ekiz H. (2004). Sliding Friction and Wear Behaviour of Polytetrafluoroethylene and its Composites under Dry Conditions. Mater. Des. 25, 239–245. 10.1016/j.matdes.2003.10.009

[B20] Wang P. Qiao G. Guo Y. Zhang Y. Hou D. Jin Z. Zhang J. Wang M. Hu X. (2020). Molecular Dynamics Simulation of the Interfacial Bonding Properties between Graphene Oxide and Calcium Silicate Hydrate. Construction Building Mater. 260, 119927. 10.1016/j.conbuildmat.2020.119927

[B21] Wang Y. Yan F. (2006). Tribological Properties of Transfer Films of PTFE-Based Composites. Wear 261, 1359–1366. 10.1016/j.wear.2006.03.050

[B22] Xie G. Y. Zhuang G. S. Sui G. X. Yang R. (2010). Tribological Behavior of PEEK/PTFE Composites Reinforced with Potassium Titanate Whiskers. Wear 268, 424–430. 10.1016/j.wear.2009.08.032

[B23] Yang S. Kwon S. Lee M. Y. Cho M. (2019). Molecular Dynamics and Micromechanics Study of Hygroelastic Behavior in Graphene Oxide-Epoxy Nanocomposites. Composites Part B: Eng. 164, 425–436. 10.1016/j.compositesb.2019.01.059

[B24] Ye J. Khare H. S. Burris D. L. (2013). Transfer Film Evolution and its Role in Promoting Ultra-low Wear of a PTFE Nanocomposite. Wear 297, 1095–1102. 10.1016/j.wear.2012.12.002

[B25] Yeo S. M. Polycarpou A. A. (2014). Fretting Experiments of Advanced Polymeric Coatings and the Effect of Transfer Films on Their Tribological Behavior. Tribology Int. 79, 16–25. 10.1016/j.triboint.2014.05.012

[B26] Zhang H.-j. Zhang Z.-z. Guo F. Jiang W. Liu W.-m. (2009). Study on the Tribological Behavior of Hybrid PTFE/cotton Fabric Composites Filled with Sb2O3 and Melamine Cyanurate. Tribology Int. 42, 1061–1066. 10.1016/j.triboint.2009.03.002

[B27] Zuo Z. Song L. Yang Y. (2015a). Tribological Behavior of Polyethersulfone-Reinforced Polytetrafluoroethylene Composite under Dry Sliding Condition. Tribology Int. 86, 17–27. 10.1016/j.triboint.2015.01.019

[B28] Zuo Z. Yang Y. Qi X. Su W. Yang X. (2014). Analysis of the Chemical Composition of the PTFE Transfer Film Produced by Sliding against Q235 Carbon Steel. Wear 320, 87–93. 10.1016/j.wear.2014.08.019

[B29] Zuo Z. Yang Y. Song L. (2015b). Miscibility Analysis of Polyethersulfone and Polytetrafluoroethylene Using the Molecular Dynamics Method. Fibers Polym. 16, 510–521. 10.1007/s12221-015-0510-2

